# 嵌合抗原受体T细胞治疗多发性骨髓瘤中国血液临床专家共识（2022年版）

**DOI:** 10.3760/cma.j.issn.0253-2727.2022.04.001

**Published:** 2022-04

**Authors:** 中国医师协会血液科医师分会 中华医学会血液学分会

嵌合抗原受体T细胞（chimeric antigen receptor T cells，CAR-T细胞）疗法是指通过基因修饰技术将带有特异性抗原识别结构域及T细胞激活信号的遗传物质转入T细胞，使T细胞直接与肿瘤细胞表面的特异性抗原结合而被激活、增殖，从而发挥靶向杀伤肿瘤细胞的作用。2016年Ali等[1]首次报道抗B细胞成熟抗原（B cell maturation antigen，BCMA）CAR-T细胞应用于复发难治性多发性骨髓瘤（RRMM），证实其有效性和安全性。随后国内外开展了大量抗BCMA CAR-T细胞治疗RRMM的临床研究，总体有效率为73％～100％（表1）[2]–[13]，部分研究中严格意义的完全缓解（sCR）/完全缓解（CR）率超过50％，其中微小残留病（MRD）1个月转阴率最高超过80％。2021年3月27日，FDA批准首个以BCMA为靶点的CAR-T细胞用于治疗RRMM。除了单靶点CAR-T细胞外，两个抗原靶点联合的CAR-T细胞临床试验有BCMA/CD19[7]、BCMA/CD38[10]、BCMA/CD138等。除了抗BCMA CAR-T外，CS1、GPRC5D、CD38、CD138等许多其他靶点的CAR-T也已进入临床试验。

CAR-T细胞治疗多发性骨髓瘤（MM）与其他B细胞肿瘤有相似的细胞因子释放综合征（cytokine release syndrome，CRS）发生率，超过80％的患者发生3级及以上血液学毒性和几乎100％的免疫学毒性；但重度CRS和免疫效应细胞相关神经毒性综合征（immune effector cell-associated neurotoxicity syndrome，ICANS）的发生率较其他B细胞肿瘤低。此外，MM常合并肾功能损害，并可合并心肌淀粉样变性，给CAR-T细胞治疗的安全性带来新的挑战。

考虑到CAR-T细胞治疗MM的快速发展和安全性，规范化的管理对于未来的临床试验和临床应用非常有必要。为此，国内同行专家共同编写了此共识，旨在提高临床CAR-T细胞治疗MM的管理水平。

一、CAR-T细胞治疗前的准备

1. 一般要求：接受CAR-T细胞治疗患者的一般要求：①KPS（Karnofsky，卡氏功能评分）≥50分或美国东部肿瘤协作组体能状况评分（ECOG评分）≤2分。②具有良好的心、肺、肝功能，左心室射血分数（LVEF）≥50％；ALT、AST水平<3倍正常范围上限，总胆红素<0.2 g/L；室内空气中患者的血氧饱和度≥92％。③无活动性感染。④预计生存期>12周。同时应排除：怀孕或哺乳期妇女或半年内有妊娠计划的妇女；传染性疾病（如活动性乙型病毒性肝炎、丙型病毒性肝炎或活动性结核等）；生命体征不正常以及不能配合检查者；有精神或心理疾病不能配合治疗及疗效评估者；对CAR-T细胞产品中任何一种有效成分有过敏史者；合并心、肺、脑等重要脏器明显功能障碍的患者。

2. 疾病评估：原发病评估主要包括患者骨髓瘤负荷、临床分期、髓外病变及器官受累等，具体评估参照《中国多发性骨髓瘤诊治指南（2022年修订）》[14]。尽管BCMA几乎表达于所有异常或正常浆细胞，但晚期复发患者及使用过BCMA靶点治疗者会出现BCMA抗原的丢失，建议评估BCMA表达及其他靶点（GPRC5D、CS1、CD19等）的表达情况。

二、CAR-T细胞治疗的应用指征

1. 商品化CAR-T：商品化的CAR-T（ABECMA，idecabtagene vicleucel，ide-cel）为鼠源的抗BCMA CAR-T，在美国被批准应用于既往接受过四线及以上治疗的成人RRMM，其中包括接受过免疫调节剂、蛋白酶体抑制剂及抗CD38单克隆抗体的患者。

2. 临床试验：目前开展的临床试验大多数针对RRMM（表1），其中包括伴高危细胞遗传学异常、髓外病变及浆细胞白血病的患者，但临床试验有治疗前移的趋势，尤其是高危患者，未来极有可能提前至一线，具体入组和排除标准见各临床试验注册信息。

**表1:** 国内外部分BCMA CAR-T细胞治疗多发性骨髓瘤的临床研究

作者	时间	临床注册号	例数	产品名称	共刺激分子
Brudno等[2]	2018	NCT02215967	24	CAR-BCMA	CD28
Li等[3]	2018	CHiCTR-OPC-16009113	30	anti-BCMA	CD28
Zhao等[4]	2018	NCT03090659	57	LCAR-B38M	4-1BB
Li等[5]	2019	ChiCTR1800018137	18	CT103A	4-1BB
Fu等[6]	2019	NCT03093168	46	HRAIN	4-1BB
Yan等[7]	2019	ChiCTR-OIC-17011272	21	anti-BCMA/anti-CD19	4-1BB
Fu等[8]	2021	NCT03975907、NCT03716856、NCT03302403、NCT03380039	38	CT053	4-1BB
Chen等[9]	2021	NCT03975907	14	CT053	4-1BB
Mei等[10]	2021	ChiCTR1800018143	26	Tandem CAR T（BCMA-CD38）	4-1BB
Berdeja等[11]	2021	NCT03548207	97	CARTITUDE-1	4-1BB
Munshi等[12]	2021	NCT03361748	128	KarMMa	4-1BB
Jin等[13]	2019	NCT03716856、NCT03302403、NCT03380039	24	CT053	4-1BB

作者	CAR-T细胞剂量	CRS发生率（％）	≥3级CRS发生率（％）	NTX（％）	≥3级NTX（％）	ORR（％）	sCR/CR（％）	中位EFS时间（月）	中位PFS时间（月）
Brudno等[2]	（0.3～9）×10^6^/kg	94	38	N/A	25	81	8.3	7.1	N/A
Li等[3]	（5.4～25）×10^6^/kg	97	20	3.3	N/A	93	73	N/A	N/A
Zhao等[4]	0.5×10^6^/kg	90	7	1.8	0	88	74	N/A	20
Li等[5]	（1～6）×10^6^/kg	94	22	0	N/A	100	37.5	N/A	N/A
Fu等[6]	9×10^6^/kg	30	6.8	NR	N/A	79.6	40.9	N/A	15
Yan等[7]	1×10^6^/kg	90	4.8	9.5	N/A	95	57	N/A	N/A
Fu等[8]	（50～180）×10^6^	73.7	0	N/A	2.6	92.1	78.9	N/A	22.7
Chen等[9]	（100～150）×10^6^	92.9	0	N/A	0	100	78.6	N/A	N/A
Mei等 [10]	（0.5～1）×10^6^/kg	87	17	0	0	85.2	52	N/A	17.2
Berdeja等[11]	0.75×10^6^/kg	95	4	21	9	97	67	N/A	未达到
Munshi等[12]	（150～450）×10^6^	84	5.5	18	3.1	73	33	N/A	8.6
Jin等[13]	（50～180）×10^6^	62.5	0	12.5	4.2	87.5	79.2	N/A	N/A

注：BCMA：B细胞成熟抗原；CAR-T细胞：嵌合抗原受体T细胞；CRS：细胞因子释放综合征；NTX：神经毒性；ORR：总缓解率；sCR/CR：严格意义的完全缓解/完全缓解；EFS：无事件生存；PFS：无进展生存；N/A：未报告

人源化和全人源CAR-T细胞降低了免疫原性，可能延长CAR-T细胞在体内的存留时间[15]，但目前尚无头对头比较鼠源与人源化或全人源CAR-T细胞的临床疗效研究。临床前动物实验显示，双靶点CAR-T细胞较单靶点有更强的抗骨髓瘤活性，可能克服骨髓瘤细胞异质性、抗原丢失或逃逸等与疾病复发相关的机制[16]。其他已进入临床试验的靶点未来可作为BCMA CAR-T细胞的重要补充或复发后治疗的选择。

3. CAR-T细胞治疗应用指征说明：临床试验一般要求KPS≥50分或ECOG评分≤2分，但ABECMA对体能状态无特殊要求，我们认为对于无更好治疗选择的患者，KPS和ECOG评分不是绝对禁忌。肾功能异常在RRMM中的发生率为40％～50％[17]，国内先后两项临床试验探索了肾功能异常患者接受BCMA为基础的CAR-T细胞治疗[18]–[19]，几乎所有患者治疗后肾功能均较前好转，且并未引起严重并发症，因此，肾功能异常非CAR-T细胞治疗的禁忌证，但可耐受CAR-T细胞治疗的最低肾小球滤过率和最高血肌酐界值仍需进一步探索。bb2121试验（BCMA靶点用于RRMM的临床试验）要求LVEF≥45％，但对于并发心肌淀粉样变性或者房颤的患者，即使LVEF正常，预处理期间或输注CAR-T细胞后并发心血管事件的风险增加，需要更多的关注[20]–[21]。乙型肝炎病毒防治可参照《靶向B细胞和浆细胞的CAR-T细胞治疗中防治乙型肝炎病毒再激活的中国专家共识（2021年版）》等[22]–[26]。体内有外源性植入物（如动/静脉导管、各类假体、静脉滤网等）的患者，需警惕诱发局部和全身感染。

三、淋巴细胞采集及影响因素

一般要求患者/供者HGB>80 g/L，PLT>50×10^9^/L（若因疾病进展导致贫血或血小板减少，评估患者获益与风险，HGB和PLT基线水平不是淋巴细胞采集的绝对限制因素），外周血中淋巴细胞绝对计数>500/µl或CD3^+^淋巴细胞>150/µl，淋巴细胞采集量一般为（60～600）×10^6^/kg[16]。

MM患者前期接受的治疗药物可能影响CAR-T细胞的活性，一般建议采集细胞前8周内未接受抗T细胞单克隆抗体、供者淋巴细胞输注及中枢神经系统放疗；2周内未接受过联合化疗、来那度胺、硼替佐米等治疗；1周内未应用长春新碱；72 h内未应用治疗剂量糖皮质激素[27]–[28]。鉴于前期治疗线数及部分药物可能会影响CAR-T细胞活性，对于高危或需要应用来那度胺治疗的患者，可考虑提前采集淋巴细胞以备用。对于既往接受过含苯达莫司汀或氟达拉滨治疗的患者，自体CAR-T细胞制备失败的可能性增加。

四、预处理

常用的预处理方案包括环磷酰胺联合氟达拉滨或环磷酰胺单药等（表2）。目前推荐采用氟达拉滨联合环磷酰胺预处理方案。

**表2:** 国内外部分BCMA CAR-T细胞治疗多发性骨髓瘤临床研究的预处理方案

产品名称	时间	临床注册号	预处理方案	
			氟达拉滨	环磷酰胺
CAR-BCMA[2]	2018	NCT02215967	30 mg/m^2^，−5～−3 d	300 mg/m^2^，−5～−3 d
LCAR-B38M[4]	2018	NCT03090659	无	300 mg/m^2^，−5～−3 d
CT103A[5]	2019	ChiCTR1800018137	25 mg/m^2^，−4～−2 d	20 mg/kg，−4～−2 d
HRAIN[6]	2019	NCT03093168	25 mg/ m^2^，−5～−3 d	300 mg/m^2^，−5～−3 d
anti-BCMA/anti-CD19[7]	2019	ChiCTR-OIC-17011272	30 mg/m^2^，−5～−3 d	750 mg/m^2^，−5 d
CT053[9]	2021	NCT03975907	25 mg/m^2^，−5～−3 d	300 mg/m^2^，−5～−3 d
Tandem CAR T（BCMA-CD38）[10]	2021	ChiCTR1800018143	25 mg/m^2^，−5～−3 d	250 mg/m^2^，−5～−3 d
CARTITUDE-1[11]	2021	NCT03548207	30 mg/m^2^，−5～−3 d	300 mg/m^2^，−5～−3 d
KarMMa[12]	2021	NCT03361748	30 mg/m^2^，−5～−3 d	300 mg/m^2^，−5～−3 d

注：BCMA：B细胞成熟抗原；CAR-T细胞：嵌合抗原受体T细胞

五、CAR-T细胞输注的管理

1. CAR-T细胞输注前：感染的筛查、预防和管理参见后文“七、CAR-T细胞治疗相关毒副作用管理”中“3. 感染”部分。既往有中枢神经系统疾病或并发症的患者发生神经系统不良事件的风险会增加，建议待疾病控制后再行CAR-T细胞输注，同时可口服左乙拉西坦（750 mg，每12 h 1次）等药物预防癫痫发生。

2. CAR-T细胞输注期间：预处理化疗结束后1～2 d输注CAR-T细胞，最长不宜超过7 d。商品化BCMA CAR-T ABECMA 建议的输注剂量为（300～460）×10^6^，CAR-T细胞输注的剂量与疗效和毒副作用相关，且不同的CAR-T细胞产品输注剂量差异很大[2]–[12]。鉴于CAR-T细胞来源于不同公司或实验室，具体剂量要依据各产品前期预实验的推荐剂量。CAR-T细胞输注前开始进行生命体征监测[29]，不推荐CAR-T细胞输注前予糖皮质激素预防过敏反应。

3. CAR-T细胞输注后：持续性监测生命体征至CRS症状消失，发生2级或以上CRS时，监护直至CRS分级降至≤1级或者根据患者的一般情况而定[27]–[29]。CAR-T细胞输注后患者应住院观察，建议至少在医院密切监测7～14 d[27]–[28]。发生CRS时每天进行体格检查并监测血常规、生化指标和凝血功能、血气分析、血清C反应蛋白、铁蛋白、白细胞介素−6等，常规监护下至少每4 h评估1次生命体征[7], [27]–[28],[30]。

六、疗效评估

CAR-T细胞治疗后采用国际骨髓瘤工作组（IMWG）和《中国MM诊治指南（2022年修订）》[14]进行疗效评估。CAR-T细胞治疗MM时，大部分对治疗有效的患者1个月内获得骨髓MRD转阴（流式细胞术）[7],[12],[31]，但免疫固定电泳转阴或轻链比例正常需要更长时间，部分患者甚至在6个月时才能达到最佳疗效[7]。详见“十、随访”。

七、CAR-T细胞治疗相关毒副作用管理

1. CRS：CRS的评估建议采用ASTCT评估标准[32]。MM患者CAR-T细胞治疗后CRS的发生率可高达90％，其中3～5级CRS发生率为5％左右[2]–[11]。CRS的临床表现多样，与受累的组织器官有关，其中发热是常见的首发表现。如果3周内出现以下4种症状或体征之一，即应考虑CRS：①发热，体温≥38 °C；②低血压，收缩压<90 mm Hg（1 mm Hg＝0.133 kPa）；③动脉血氧饱和度<90％；④出现器官毒性[29]。鉴于以上均为非特异性临床表现，诊断CRS必须排除其他并发症，包括感染、肿瘤溶解综合征及过敏反应等[32]。CRS也可迟至4周后发生，对有迟发CRS表现者，仍要警惕。托珠单抗和糖皮质激素是治疗严重或危及生命CRS的主要药物，可根据不同CAR-T细胞产品的安全性推荐，适时应用托珠单抗和（或）糖皮质激素（包括一线应用），在症状消失之前，应密切监测CRS患者的心脏和其他器官功能，同时考虑重症监护和积极支持治疗。对于一线干预后12 h内发热、终末器官毒性（如缺氧、低血压）等无改善者，需警惕并发噬血细胞性淋巴组织细胞增生症/巨噬细胞活化综合征（HLH/MAS）。CRS的分级管理见表3、表4，具体参照CAR-T细胞治疗相关毒性评估和管理建议[29],[32]。

**表3:** 细胞因子释放综合征（CRS）分级评估指标及依据

CRS分级	评估指标及依据
1级	发热（体温>38°C，伴或不伴其他体征），且排除其他发热原因
2级	发热伴低血压（不需应用升压药）和（或）低血氧（需要低流量吸氧）
3级	发热伴低血压（需要一种或不需应用升压药）和（或）低血氧（需要高流量鼻导管、面罩吸氧，但不需要借助机械通气）
4级	发热伴低血压（需要多种升压药，但不包括血管加压素）和（或）低血氧（需正压通气，包括持续气道正压通气、气管插管和机械通气）

**表4:** 细胞因子释放综合征（CRS）分级管理建议

CRS分级	治疗措施
1级	密切监护，支持治疗，评估感染，检测体液平衡，按需使用解热镇痛药物
2级	密切监护，支持治疗，监测心脏和其他脏器功能，老年或合并并发症的患者可使用托珠单抗和（或）糖皮质激素
3级	密切监护，支持治疗，使用托珠单抗和（或）糖皮质激素（在托珠单抗静脉输注1～2次后低血压持续存在时，地塞米松可以每6 h 10 mg静脉滴注，如为难治性，增加至每6 h 20 mg静脉滴注）
4级	密切监护，支持治疗，使用托珠单抗和（或）糖皮质激素，可予甲泼尼龙1 g/d静脉输注

注：托珠单抗用法为8 mg/kg（单次剂量不超过800 mg），静脉滴注时间大于1 h，控制不佳者可8 h后再次使用，24 h内不超过4次，总次数不得超过4次

2. 神经毒性：MM患者CAR-T细胞治疗后神经毒性的发生率为10％～42％，其中3级及以上的发生率为1％～3％[32]。神经毒性表现多样，评估项目除意识、语言、书写外，根据需要及时行脑电图检查、眼底检查（评估视乳头水肿）、脑部影像学检查、腰椎穿刺术（测定颅内压）等 [29],[32]；ICANS根据ICE积分、意识状态、颅内压升高/脑水肿、癫痫或运动能力下降情况分为1～4级[32]，ICANS的分级管理见表5。另外，CAR-T细胞可能诱发一些特殊的神经毒性（如帕金森样表现）。

**表5:** 免疫效应细胞相关神经毒性综合征（ICANS）的分级管理

ICANS分级	未并发CRS	并发CRS的附加治疗
1级	支持治疗	托珠单抗8 mg/kg，静脉输注
2级	支持治疗 地塞米松10 mg，每6 h 1次，静脉滴注；或甲泼尼龙1 mg/kg，每12 h 1次，静脉滴注	托珠单抗8 mg/kg，静脉输注 若并发≥2级CRS，考虑将患者转移至ICU
3级	建议转移至ICU 支持治疗 地塞米松10 mg，每6 h 1次，静脉滴注；或甲泼尼龙1 mg/kg，每12 h 1次，静脉输注 若ICANS持续≥3级，每2～3 d重复进行神经影像学（CT或MRI）检查	托珠单抗8 mg/kg，静脉输注
4级	支持治疗 ICU监护，建议机械通气 大剂量激素（甲泼尼龙1 g/d），静脉输注 若ICANS持续≥3级，每2～3 d重复进行神经影像学（CT或MRI）检查 参照指南治疗惊厥性癫痫持续状态患者	支持治疗 ICU监护，建议机械通气 大剂量激素（甲泼尼龙1 g/d），静脉输注 若ICANS持续≥3级，每2～3 d重复进行神经影像学（CT或MRI）检查 参照指南治疗惊厥性癫痫持续状态患者

注：CRS：细胞因子释放综合征

3. 感染：感染是CAR-T细胞治疗中的重要并发症[32]，常与CRS同时发生，病原体包括细菌、病毒、真菌、支原体及其他。Wang等[33]观察BCMA CAR-T细胞治疗MM时，57.4％的患者并发感染，以下呼吸道为主（53％），其次为上呼吸道感染（21％）。病原体依次为细菌（57％）、病毒（18％）、真菌（16％）、支原体（7％）及其他（2％）。CAR-T细胞治疗前需全面筛查（包括但不限于人类免疫缺陷病毒、丙型肝炎病毒、乙型肝炎病毒、巨细胞病毒、结核分枝杆菌等）并控制活动性感染。预处理开始时予阿昔洛韦或伐昔洛韦，用药时间根据免疫功能恢复情况确定。输注CAR-T细胞至粒细胞恢复前给予诺氟沙星、三唑类抗真菌药及复方磺胺甲唑片等预防感染，其中卡氏肺囊虫感染预防至少3个月。感染治疗可参考《中国中性粒细胞缺乏伴发热患者抗菌药物临床应用指南（2016年版）》进行管理[34]；发生3～4度粒细胞缺乏时，有条件者可隔离保护，同时应用粒细胞集落刺激因子（G-CSF）[27]–[29],[34]。

4. B细胞缺乏和低免疫球蛋白血症：低免疫球蛋白血症是MM患者CAR-T细胞治疗后常见的并发症，BCMA单靶点治疗时几乎所有有效的患者发生低免疫球蛋白血症和B细胞缺乏。B细胞恢复至基线的中位时间为79 d，1年时IgG、IgA及IgM恢复至正常水平的比例分别为53.33％、23.81％及73.08％。BCMA/CD19双靶点治疗时，低免疫球蛋白血症和B细胞缺乏发生率为100％，其中B细胞一般于CAR-T细胞输注后2个月逐渐恢复，IgM于3个月后开始恢复，而低IgA和IgG持续时间甚至超过1年[3],[35]，可能增加感染机会。CAR-T细胞治疗后3个月内应每月监测患者血清IgG水平，血清IgG<4 g/L者或血清IgG 4～6 g/L且并发感染者，应用丙种球蛋白替代治疗；血清IgG>6 g/L且并发感染者，建议进一步评估各型免疫球蛋白水平（IgG、IgA及IgM）和B细胞数量。

5. 重要脏器损伤：CRS发生过程中常伴重要脏器功能受累和损伤，包括心脏、消化道、肝脏、肌肉、胰腺等，其中心脏受损和消化道出血风险最大[36]。具体可参照NCCN免疫治疗相关毒性管理[36]。

6. 骨髓抑制：MM患者CAR-T细胞治疗较其他B细胞肿瘤有更严重的骨髓抑制，KarMMa研究显示[12]，3～4级粒细胞、血小板减少和贫血的发生率分别为96％、63％、63％，应积极行对症和支持治疗。

7. 其他：其他治疗相关不良反应包括凝血功能异常、肿瘤溶解综合征、HLH/MAS等，具体可参照NCCN免疫治疗相关毒性管理[36]和CAR-T细胞治疗毒性评估和管理[29]。

八、CAR-T细胞治疗后维持治疗

复发已成为RRMM患者接受CAR-T细胞治疗后面临的主要挑战之一，CAR-T细胞治疗RRMM的中位PFS时间为8.8～11.8个月，疗效最好的中心中位PFS时间为22.7个月[12]，获得sCR或CR的患者1年持续缓解率约70％[4]。因此，CAR-T细胞治疗后复发是有待解决的难题之一，但到目前为止，CAR-T细胞治疗后是否应维持治疗或如何维持无基于循证医学的证据。维持治疗是CAR-T细胞未来一段时间重点探索的方向之一，包括造血干细胞移植、免疫调节剂、纠正免疫微环境等。

九、复发或进展后挽救性治疗

CAR-T细胞治疗后复发的治疗可参照《中国多发性骨髓瘤诊治指南（2022年修订）》[14]，包括临床试验、既往敏感的药物和未使用的新药或新的联合治疗方案。二次CAR-T细胞治疗也值得临床尝试，但需要基于以下考虑：①靶抗原阳性复发的患者，如首次接受鼠源性BCMA CAR-T复发后可选择人源化或全人源BCMA CAR-T或双靶点联合，也可选择其他可检测到的靶点；②靶抗原表达降低复发可选择联合γ分泌酶抑制剂[37]；③对靶抗原阴性复发的患者，只能选择可检测到的其他靶抗原；④更换不同共刺激分子的CAR-T细胞[38]；⑤对于CAR-T细胞在体内持续时间短或扩增差的患者，首先评估患者T细胞和CAR-T细胞功能或是否存在影响T细胞活性的因素[39]，可延长洗脱期或尝试非自体CAR-T细胞。

十、随访

随访应包括以下三个方面内容：原发病持续缓解情况、远期不良反应及感染的防治。CAR-T细胞治疗后14 d和28 d，半年内每月评估一次，6～12个月每2个月评估一次，主要评估疾病的缓解状况和不良反应；第二年每3个月进行一次全面评估；第三年（及以后），每3～6个月或根据临床情况进行全面评估。评估的指标包括M蛋白定量、血清蛋白电泳、免疫固定电泳及游离轻链检测、骨髓细胞学、MRD；有髓外病变患者还需完善相关影像学检查，包括皮肤、软组织、淋巴结、肝、脾及中枢神经系统MRI，必要时行PET-CT检查。伴髓外病变者，建议在CAR-T细胞治疗1个月后评估髓外病变，MRI、CT或X线均可作为评估手段，3个月后可考虑PET-CT评估。对于考虑疾病进展的患者应立即予以评估。感染防治参见“七、CAR-T细胞治疗相关毒副作用管理”中感染防治和低免疫球蛋白血症管理。此外，所有接受以病毒为载体制备CAR-T细胞治疗的患者，均需监测远期生物安全性。
